# Effect of equine-derived *Lactobacillus* M11 on the reproductive performance of KM pregnant female mice

**DOI:** 10.3389/fmicb.2026.1741988

**Published:** 2026-02-02

**Authors:** Yuanyi Liu, Haoran Xu, Jialong Cao, Qianqian He, Na Wang, Ming Du, Yiping Zhao, Manglai Dugarjaviin, Xinzhuang Zhang

**Affiliations:** 1 Key Laboratory of Equus Germplasm Innovation, Ministry of Agriculture and Rural Affairs, Hohhot, China; 2 Inner Mongolia Key Laboratory of Equine Science Research and Technology Innovation, Inner Mongolia Agricultural University, Hohhot, China; 3 College of Animal Science, Inner Mongolia Agricultural University, Hohhot, China

**Keywords:** equine-derived *Lactobacillus* M11, metabolomic profiling, microbial community reconfiguration, pregnant kunming mice, reproductive performance

## Abstract

**Introduction:**

This study aimed to evaluate the effects of equine-derived *Lactobacillus* M11 on reproductive performance and metabolic profiles in pregnant Kunming (KM) mice. The objective was to explore the potential of M11 as a safe and effective alternative to antibiotics in antibiotic-free farming systems.

**Methods:**

Specific pathogen-free (SPF) female KM mice were randomly assigned to a blank control group (BC) and three intervention groups (M11-L, M11-M, M11-H). The intervention groups received daily gavage of M11 at low (1.0 × 10^7^ CFU/mL), medium (1.0 × 10^8^ CFU/mL), and high (1.0 × 10^9^ CFU/mL) concentrations for 21 days. Host physiological parameters, metagenomic profiles, and metabolomic signatures were analyzed to assess the impact of M11 supplementation.

**Results:**

(1) Host Physiology and Biochemistry: The M11-H group exhibited a significant elevation in albumin (ALB; 40.30 ± 1.75 g/L), suggesting enhanced nutritional status or hepatic protein synthesis. The M11-L group showed transient increases in alanine aminotransferase (ALT; 59.57 ± 10.34 U/L) and total cholesterol (TC; 2.90 ± 0.24 mmol/L), indicative of adaptive hepatic lipid metabolism. (2) Microbial Community Reconfiguration: Metagenomic analysis revealed significant structural shifts in the gut microbiota between the BC and M11-H groups. Notably, the M11-H group showed enrichment of Bacillota, which correlated with “O-antigen nucleotide sugar biosynthesis,” while differences in Pseudomonadota were associated with immune regulation. (3) Metabolomic Profiling: Partial Least Squares Discriminant Analysis (PLS-DA) demonstrated clear separation in the cecal metabolome space. KEGG pathway enrichment analysis highlighted significant alterations in “glycine/serine/threonine metabolism” and “arginine/proline metabolism” pathways. (4) Integrated Multi-Omics Analysis: Correlation analysis identified a significant positive association between *s_Clostridiaceae_bacterium* (Bacillota) and specific metabolites (3-hydroxy-4-aminopyridine sulfate), suggesting the formation of a regulatory “gut-reproductive axis.”

**Discussion:**

The results demonstrate that *Lactobacillus* M11 improves metabolic support during pregnancy through three primary mechanisms: modulation of the gut microbiota, activation of key metabolic pathways, and enhancement of antioxidant capacity. These findings provide a theoretical basis for the application of probiotic-mediated reproductive support in antibiotic-free farming, highlighting M11 as a promising candidate for improving livestock health and productivity.

## 1 Introduction

In animal husbandry, among the various microorganisms used as probiotics, *Lactobacillus*, a specific group within the lactic acid bacteria, represents the most extensively utilized category. As an important probiotic group, *Lactobacillus* exerts positive effects on host health through multiple mechanisms, including regulating the host’s microecological balance, enhancing immune function, and promoting nutrient absorption (Hashem and Gonzalez-Bulnes, 2022). Among them, *Lactobacillus salivarius*, as a typical probiotic strain, not only has good colonization ability and acid tolerance but can also secrete bacteriocins to inhibit the growth of pathogenic bacteria (Pereira et al., 2022).

Reproductive performance is a key determinant of the quality of animal populations and production efficiency, influenced by multiple factors including genetics, hormonal regulation, and the nutritional environment (Hashem and Gonzalez-Bulnes, 2022). In animal production, regulating reproductive efficiency is of utmost importance for optimizing population genetic progress and economic benefits. The maternal pregnancy rate, litter size, and offspring survival rate directly affect the speed of population expansion, thereby impacting the economic returns of major breeding farms. In recent years, significant progress has been made in research on probiotics in the field of animal reproductive health. Studies have demonstrated that probiotics hold great potential in maintaining and restoring the ecological balance of the reproductive system and can serve as a safe alternative to antibiotics for improving animal reproductive function (Hashem and Gonzalez-Bulnes, 2022). An imbalance in the reproductive tract microbiota is closely associated with various reproductive diseases and disorders in mammals. Probiotics, with their antibacterial, antiviral, and immunomodulatory properties, offer new therapeutic strategies for maintaining reproductive ecological balance (Abbasi et al., 2022). In pig production, probiotics have been shown to enhance reproductive performance during the gestation, parturition, and lactation periods of sows (Pereira et al., 2022).

Equine - derived *Lactobacillus*, as a novel probiotic resource, has a unique biological origin. Horses, as non - ruminant herbivores, have a distinct digestive system and microbial ecosystem compared to common livestock animals. The *Lactobacillus* strains derived from horses may have adapted to this specific environment and developed unique metabolic characteristics and functional properties (Zhao et al., 2025). These characteristics might enable them to interact with the host’s reproductive system in a distinct way, potentially influencing reproductive performance through mechanisms such as modulating the reproductive tract microbiota, enhancing immune responses in the reproductive organs, or affecting the synthesis and metabolism of reproductive - related hormones. Therefore, further in - depth research on the mechanisms and effects of equine - derived *Lactobacillus* in improving animal reproductive performance is warranted (Yáñez Ramil et al., 2025).

Therefore, this study aims to investigate the impact of equine-derived *Lactobacillus* on the reproductive performance of KM pregnant female mice, providing a theoretical basis for the development of novel probiotic preparations and offering new solutions for addressing animal reproduction issues under antibiotic-free breeding conditions.

## 2 Materials and methods

### 2.1 Headings ethics statement

This study was approved by the Ethics Committee of the Inner Mongolia Agricultural University (No. NND2024009). All experimental procedures adhered to the guidelines for the care and use of experimental animals.

To euthanize mice in an ethical and humane manner, we employ a well - established protocol. First, the mice are carefully handled and placed in a clean, quiet, and appropriately sized chamber. Carbon dioxide (CO_2_) inhalation is the chosen method, which is widely recognized as a humane euthanasia technique for small rodents. The chamber is gradually filled with CO_2_ at a controlled rate, typically not exceeding 30% of the chamber volume per minute. This slow introduction of CO_2_ allows the mice to gradually lose consciousness without experiencing undue stress or pain. The mice are continuously monitored during the process. Once the CO_2_ concentration reaches a sufficient level to ensure rapid loss of consciousness and subsequent death (usually confirmed by the cessation of breathing and lack of response to stimuli), the CO_2_ flow is maintained for an additional period, often around 1–2 min, to ensure complete euthanasia. After this, the mice are removed from the chamber and their death is further verified by checking for the absence of a heartbeat and other vital signs. All steps are carried out by trained personnel who are well - versed in the proper handling and euthanasia procedures of mice, and the entire process is documented in detail for ethical and regulatory compliance (Boivin et al., 2017).

### 2.2 Preparation and grouping of experimental strains

A strain of *Lactobacillus* M11 was previously isolated by our laboratory. From a taxonomic perspective, this strain belongs to the species *Lactobacillus salivarius* (Zhao et al., 2025). The following is a detailed description of the preparation and grouping of experimental strains.

#### 2.2.1 Sample collection

In this research, the sampling population comprised female Mongolian horses, aged between 2 and 3 years, sourced from the Salaqi Horse Farm located in Baotou City, Inner Mongolia. A comprehensive veterinary examination was conducted to ascertain that all horses were in good health and free from any diseases. Subsequently, the vulva of each mare was meticulously cleaned before collecting vaginal secretions with the aid of sterile vaginal swabs. These secretions were promptly transferred into sterile anaerobic bags, transported in a refrigerated container, and ultimately processed within a sterile laminar flow hood at the laboratory of Inner Mongolia Agricultural University (Zhao et al., 2025).

#### 2.2.2 Isolation and purification

Swab specimens were immersed in 50 mL of phosphate-buffered saline (PBS), thoroughly vortexed, and then subjected to a series of dilutions ranging from 10^−1^ to 10^−6^. Portions (200 μL) of the 10^−4^, 10^−5^, and 10^−6^ dilutions were evenly distributed across MRS agar plates supplemented with bromocresol purple (0.04 g/L). The MRS Broth (Qingdao Hopebio, HB0384-1) and Bromocresol Purple Agar (Qingdao Hopebio, HB8616-2) utilized in this procedure were procured from commercial vendors. Subsequently, the plates were incubated under anaerobic conditions at 37 °C for a duration of 24 to 36 h (Zhao et al., 2025).

Colonies exhibiting a yellow hue, a characteristic indicator of lactic acid production, were streaked onto fresh MRS agar plates and further subcultured in MRS broth at 37 °C for 18 to 24 h. These cultures were then preserved by storing them in a solution of 20% glycerol at a temperature of −80 °C (Zhao et al., 2025).

#### 2.2.3 Grouping of experimental strains

The pre-prepared MRS (Qingdao Haibo Biotechnology Co., Ltd.) broth medium, which had undergone high-pressure sterilization, was taken out and placed in a laminar flow hood for operation. One milliliter of the preserved strain M11 was added to 100 mL of the MRS broth medium and thoroughly mixed. The mixture was then placed in a constant-temperature shaker (at 37 °C) for activation under anaerobic conditions for 8 to 12 h. The concentration of the bacterial suspension was determined when the optical density (OD) value, measured using the spread plate method and a microplate reader, reached 1.0. The bacterial suspension was subsequently prepared into three concentration groups: 1.0 × 10^7^ CFU/mL, 1.0 × 10^8^ CFU/mL, and 1.0 × 10^9^ CFU/mL, for subsequent experimental use (Zhao et al., 2025).

### 2.3 Experimental animals and sample collection

A total of 40 five-week-old specific pathogen-free (SPF) female Kunming (KM) mice, with a body mass of (20 ± 2) g, were purchased from Inner Mongolia Calvin Biotechnology Co., Ltd. The mice were allowed ad libitum access to food and water. After a 7-day acclimation period, the mice were randomly assigned to four groups, with 10 mice in each group. The groups were as follows: the blank control group (BC), the low-concentration (1.0 × 10^7^ CFU/mL) group (M11-L), the medium-concentration (1.0 × 10^8^ CFU/mL) group (M11-M), and the high-concentration (1.0 × 10^9^ CFU/mL) group (M11-H).

The experimental period spanned 21 days, during which gavage administration was performed. Mouse body weight was recorded every 7 days. Notably, as days 14 to 21 constituted the delivery period for the mice, no body weight recordings were made during this interval. Consequently, body weight and feed intake were recorded at days 0, 7, and 14. Specifically, the daily feed consumption of each mouse was recorded by weighing the provided feed at the beginning of each day and then weighing the remaining feed at the end of the day. The feed intake of mice is presented in Supplementary Table 1. In the blank control group (BC), mice were gavaged with 0.6 mL of sterile saline per day. In the low-concentration group (M11-L), mice received a daily gavage of 0.6 mL of an M6 bacterial suspension at a concentration of 1.0 × 10^7^ CFU/mL (Gao et al., 2022). In the medium-concentration group (M11-M), mice were gavaged daily with 0.6 mL of an M11 bacterial suspension at a concentration of 1.0 × 10^8^ CFU/mL. In the high-concentration group (M11-H), mice were gavaged daily with 0.6 mL of an M6 bacterial suspension at a concentration of 1.0 × 10^9^ CFU/mL (Li et al., 2020).

At the end of the experiment, mice that were not pregnant or died during the course were excluded. The remaining mice in each group were as follows: the blank control group (BC, 6 mice), the low-concentration group (M11-L, 7 mice), the medium-concentration group (M11-M, 6 mice), and the high-concentration group (M11-H, 9 mice). After fasting for 8 to 12 h, the mice were euthanized with carbon dioxide and dissected. Samples of the liver, both kidneys, spleen, and intestine were rapidly collected from each mice for subsequent experiments. The weights of mice organs (liver, both kidneys, spleen and intestine,) are presented in Supplementary Table 2. The heart, positioned slightly to the left of the central region, is punctured with a needle of a 5 - mL syringe to draw blood. After blood collection, the blood sample is transferred into a sterile centrifuge tube and allowed to stand at room temperature for 30 min to facilitate clotting. Subsequently, the tube is centrifuged at a speed of 2000 × g for 15 min. The clear, yellowish supernatant, which is the serum, is then carefully aspirated using a pipette and transferred into a new sterile tube for subsequent experimental use.

### 2.4 Determination of litter weight and number of offspring in mice

After the experiment concluded, the litter weight of the experimental mice in each group was accurately weighed. A precision electronic balance was employed during the weighing process to guarantee data accuracy. The litter weight data for each group were recorded, and the number of offspring born to the experimental mice in each group was also carefully documented.

### 2.5 Determination of blood biochemical indicators

The concentrations of Albumin (ALB, g/L), Total Protein (TP, g/L), Globulin (GLOB, g/L), Total Bilirubin (TB), Aspartate Aminotransferase (AST, U/L), Alanine Aminotransferase (ALT, U/L), Alkaline Phosphatase (ALP, U/L), Lipase (LPS, U/L), Lactate Dehydrogenase (LDH, U/L), Creatine Kinase (CK, U/L), Creatinine (Crea, μmol/L), Uric Acid (UA, μmol/L), Blood Urea Nitrogen (BUN, mmol/L), Total Cholesterol (TC, mmol/L), and Triglyceride (TG, mmol/L) were measured using a biochemical analyzer (Instrument version number: V1.00.01.20/1.00.01.55) at Ruipai Animal Hospital, Inner Mongolia Agricultural University.

### 2.6 Determination of immunological indicators

Retrieve the previously frozen blood samples for the examination of immunological indicators. The immunological indicators interleukin - 6 (IL - 6), immunoglobulin A (IgA), immunoglobulin G (IgG), and immunoglobulin M (IgM) were measured using enzyme - linked immunosorbent assay (ELISA) detection kits. The experimental operations strictly adhered to the guidance steps provided in the kit manuals. The following ELISA kits were all purchased from Jiangsu Meibiao Biotechnology Co., Ltd.: MB - 2899A: Mouse Interleukin - 6 (IL - 6) ELISA Research Kit (96 - well plate); MB - 2791A: Mouse Immunoglobulin A (IgA) ELISA Research Kit (96 - well plate); MB - 2791B; MB - 2793A; Mouse Immunoglobulin G (IgG) ELISA Research Kit (96 - well plate); MB - 2794A: Mouse Immunoglobulin M (IgM) ELISA Research Kit (96 - well plate).

### 2.7 Determination of antioxidant indicators

Retrieve the previously frozen blood samples for the examination of antioxidant indicators. The antioxidant indicators, namely superoxide dismutase (SOD), malondialdehyde (MDA), glutathione peroxidase (GSH-PX), total antioxidant capacity (T-AOC), and catalase (CAT), were measured using the following assay kits. All kits were purchased from Nanjing Jiancheng Bioengineering Institute, and the operations strictly adhered to the instructions provided in the reagent manuals: Superoxide Dismutase (SOD): Total Superoxide Dmutase (SOD) Assay Kit (WST - microplate method), product number A001-3; Malondialdehyde (MDA): Malondialdehyde (MDA) Assay Kit (TBA method), product number A003-1-2; Glutathione Peroxidase (GSH - PX): Glutathione Peroxidase (GSH - PX) Assay Kit, product number A005-1-2; Total Antioxidant Capacity (T - AOC): Total Antioxidant Capacity (T - AOC) Assay Kit (ABTS method), product number A015-2-1; Catalase (CAT): Catalase (CAT) Assay Kit (visible spectrophotometry), product number A007-1-1.

### 2.8 Localization of *Lactobacillus* M11 in the mice intestinal tract

In the experiment, RNA was extracted from the jejunum, ileum, cecum, and colon tissues of each group of mice obtained through the aforementioned procedures, followed by reverse transcription into cDNA. Subsequently, real-time fluorescent quantitative PCR was performed. This facilitated the localization of *Lactobacillus* M11 within the mice’s intestinal tracts. The specific experimental procedures are as follows.

#### 2.8.1 RNA extraction and reverse transcription

Total RNA (including mRNA) was isolated from mouse jejunum, ileum, cecum, and colon tissues using the TRIzol™ Reagent (Thermo Fisher Scientific, Waltham, USA). Briefly, tissue samples (50 mg) were homogenized in 1 mL TRIzol reagent using a tissue grinder, followed by incubation at room temperature for 5 min to permit complete dissociation of nucleoprotein complexes. Chloroform (200 μL per 1 mL TRIzol) was added, and the mixture was vigorously vortexed for 15 s before centrifugation at 12,000 × g for 15 min at 4 °C. The aqueous phase containing RNA was transferred to a new tube, and RNA was precipitated with an equal volume of isopropanol at −20 °C for 30 min. After centrifugation at 12,000 × g for 10 min at 4 °C, the RNA pellet was washed twice with 75% ethanol, air-dried, and dissolved in 20 μL RNase-free water.

The extracted RNA (1 μg per sample) was reverse transcribed into cDNA using the PrimeScript™ RT Master Mix (Perfect Real Time) kit (TaKaRa Bio Inc., Dalian, China) in a 20 μL reaction system (Supplementary Table 3). Reverse transcription was performed at 37 °C for 15 min followed by inactivation at 85 °C for 5 s. The resulting cDNA was stored at −20 °C until further use.

#### 2.8.2 Real-time quantitative PCR

Primers were designed using Primer (5.0) software based on NCBI reference sequences (accessed on December 14, 2025, at https://www.ncbi.nlm.nih.gov) and synthesized by Sangon Biotech Co., Ltd. (Shanghai, China). *β-actin* was selected as the internal reference gene for real-time quantitative PCR, with three technical replicates performed (Vázquez-Carretero et al., 2014; Vázquez-Carretero et al., 2016). Real-time quantitative PCR was conducted using a fluorescence quantitative PCR detection system (BIO-RAD, Hercules, California, USA). The relative gene expression levels were calculated using the 2^−∆∆Ct^ method. Supplementary Table 4 records the primer information, Supplementary Table 5 records the reaction system for real-time quantitative PCR, and Supplementary Table 5 records the sequence information of *Lactobacillus* M11.

### 2.9 Experimental and analytical methods for metagenomics

Metagenomic profiling was conducted on the cecal contents of mice from the blank control group (BC) and the high-concentration group (M11-H).

#### 2.9.1 DNA extractions

DNA was extracted from various mouse fecal samples using the cetyltrimethylammonium bromide (CTAB) method, with meticulous adherence to the manufacturer’s instructions. To safeguard the accuracy and reliability of the experimental outcomes, negative controls were integrated into the study. These controls comprised unused swabs that were subjected to the entire DNA extraction workflow. Following extraction, these controls were rigorously tested and verified to be free of any DNA amplicons, ensuring the absence of contamination. The total DNA obtained was eluted into 50 μL of elution buffer, employing a modified version of the protocol recommended by the manufacturer (QIAGEN). Subsequently, the eluted DNA samples were stored at −80 °C until they were ready for PCR analysis. The PCR analysis was performed by LC-BIO Technologies (Hangzhou) Co., Ltd., situated in Hangzhou, Zhejiang Province, China.

#### 2.9.2 DNA library construction

The DNA library was assembled utilizing the Fast DNA Library Prep Set for Illumina (CW3045M). Initially, the DNA underwent fragmentation via dsDNA Fragmentase (NEB, M0348S), achieved by incubating the samples at 37 °C for 30 min. The library construction process commenced with these fragmented cDNA molecules. To generate blunt-end DNA fragments, a combination of fill-in reactions and exonuclease activity was employed. Subsequently, size selection of the DNA fragments was carried out using the sample purification beads provided in the kit. Following size selection, an adenine (A) base was appended to the blunt ends of each DNA strand, rendering them suitable for ligation to indexed adapters. Each adapter featured a thymine (T) base overhang, facilitating the ligation of the adapter to the A-tailed, fragmented DNA. These adapters were designed to encompass the complete set of sequencing primer hybridization sites necessary for single, paired-end, and indexed reads. Depending on the experimental requirements, either single- or dual-index adapters were ligated to the DNA fragments. The ligated products were then subjected to PCR amplification under the following conditions: an initial denaturation step at 95 °C for 3 min, followed by 8 cycles of denaturation at 98 °C for 15 s, annealing at 60 °C for 15 s, and extension at 72 °C for 30 s. The PCR amplification concluded with a final extension step at 72 °C for 5 min.

#### 2.9.3 Data analysis

The raw sequencing reads underwent a series of processing steps to yield high-quality reads suitable for subsequent analysis. Initially, sequencing adapters were excised from the reads using cutadapt version 1.9. Subsequently, reads of low quality were trimmed employing fqtrim version 0.94, which utilized a sliding-window algorithm for precise trimming. To eliminate host-derived contamination, the reads were aligned against the host genome using bowtie2 version 2.2.0. Once the reads had undergone quality filtering, they were subjected to *de novo* assembly using MEGAHIT version 1.2.9, thereby constructing a metagenome for each individual sample. The coding regions (CDS) within the metagenomic contigs were then predicted using MetaGeneMark version 3.26. The CDS sequences from all samples were clustered together using CD-HIT version 4.6.1 to generate unigenes. For each sample, the abundance of unigenes was estimated by calculating the Transcripts Per Million (TPM) values, based on the number of reads that aligned to each unigene using bowtie2 version 2.2.0. The taxonomic classification of the unigenes was determined by aligning them against the NCBI NR database using DIAMOND version 0.9.14, thereby identifying their lowest common ancestor taxonomy. Similarly, functional annotations of the unigenes were obtained across multiple databases. Leveraging the taxonomic and functional annotations of the unigenes, along with their abundance profiles, differential analysis was conducted at various levels—taxonomic, functional, or gene-wise. This analysis employed Fisher’s exact test for non-replicated groups and the Kruskal-Wallis test for replicated groups to discern statistically significant differences.

### 2.10 Experimental and analytical methods for untargeted metabolomics

Metabolomic profiling was conducted on the cecal contents of mice from the blank control group (BC) and the high-concentration group (M11-H).

#### 2.10.1 Metabolite extraction

The gathered samples were defrosted on ice, after which metabolites were extracted using a lipid extraction buffer. Specifically, 100 mg of each sample was subjected to extraction with 1 mL of pre-chilled lipid extraction buffer (comprising IPA: ACN: H_2_O in a ratio of 2:1:1). The mixture was vortexed for 1 min and subsequently incubated at room temperature for 10 min. Following this, the extraction mixture was stored at −20 °C overnight. After centrifugation at 4,000 g for 20 min, the supernatants were carefully transferred to new 96-well plates. Prior to LC–MS analysis, the samples were stored at −80 °C. Additionally, pooled quality control (QC) samples were prepared by combining 10 μL from each extraction mixture (Smith et al., 2006; Kuhl et al., 2012).

#### 2.10.2 Description of liquid chromatography parameters

All sample acquisitions were carried out using the LC–MS system in strict accordance with the machine’s operational protocols. Chromatographic separations were conducted utilizing an ACQUITY UPLC System (Waters, Milford, MA, USA). For reversed-phase separation, a Kinetex UPLC C18 column (100 mm × 2.1 mm, 100 Å, Phenomenex, UK) was employed. The column oven was maintained at a constant temperature of 55 °C. The flow rate was set at 0.3 mL/min. The mobile phase comprised two solvents: Solvent A, a mixture of ACN and H_2_O in a ratio of 6:4 with 0.1% formic acid, and Solvent B, a combination of IPA and ACN in a ratio of 9:1 also containing 0.1% formic acid. The gradient elution conditions were precisely defined as follows: from 0 to 0.4 min, the proportion of Solvent B was maintained at 30%; from 0.4 to 1 min, it increased linearly from 30 to 45%; from 1 to 3 min, it further rose from 45 to 60%; from 3.5 to 5 min, it went from 60 to 75%; from 5 to 7 min, it increased from 75 to 90%; from 7 to 8.5 min, it climbed from 90 to 100%; from 8.5 to 8.6 min, Solvent B remained at 100%; from 8.6 to 8.61 min, it decreased from 100 to 30%; and from 8.61 to 10 min, Solvent B was held at 30% (Wen et al., 2017; Zhou et al., 2022).

#### 2.10.3 Description of mass spectrometry parameters

A high-resolution TripleTOF 6,600 tandem mass spectrometer (SCIEX, Framingham, MA, USA) was employed for the detection of metabolites eluted from the column. The Q-TOF instrument was operated in both positive and negative ion modes. The curtain gas pressure was set at 30 PSI, while Ion source gas 1 and Ion gas 2 were both adjusted to 60 PSI. The interface heater temperature was maintained at 650 °C. In positive ion mode, the Ionspray voltage floating was set at 5000 V, whereas in negative ion mode, it was set at −4,500 V. Mass spectrometry data acquisition was performed in Information-Dependent Acquisition (IDA) mode. The TOF mass range spanned from 60 to 1,200 Da. Survey scans were acquired within 150 ms, and up to 12 product ion scans were collected provided they exceeded a threshold of 100 counts per second (counts/s) and possessed a 1 + charge-state. The total cycle time was fixed at 0.56 s. For each scan, four time bins were summed at a pulser frequency of 11 kHz, achieved through monitoring via the 40 GHz multichannel TDC detector with four-anode/channel detection. Dynamic exclusion was set for a duration of 4 s. During data acquisition, mass accuracy calibration was conducted every 20 samples. Furthermore, to assess the stability of the LC–MS system throughout the entire acquisition process, a quality control sample (a pool of all samples) was analyzed after every 10 samples (Dieterle et al., 2006; Li et al., 2022).

#### 2.10.4 Information analysis workflow

Pretreatment of the acquired mass spectrometry (MS) data, which includes peak picking, peak grouping, retention time correction, secondary peak grouping, as well as isotope and adduct annotation, was carried out using XCMS software. The raw LC–MS data files were first converted into the mzXML format and then processed via the XCMS, CAMERA, and metaX toolboxes integrated within the R software. Each ion was uniquely identified by integrating retention time (RT) and mass-to-charge ratio (m/z) data, with peak intensities recorded to generate a three-dimensional matrix comprising peak indices (retention time-m/z pairs), sample names (observations), and ion intensity information (variables). Metabolite annotation was performed by matching precise molecular mass data (m/z) of samples with those from online KEGG and HMDB databases; metabolites with a mass difference less than 10 parts per million (ppm) were annotated, and their molecular formulas were further validated through isotopic distribution measurements and an in-house fragment spectrum library. Statistical analysis, primarily conducted using R (version 4.0), involved three key steps: data filtering to remove samples with over 80% missing values or quality control samples with over 50% missing data, data imputation using the K-nearest neighbor (KNN) method, and data standardization via Probabilistic quotient normalization (PQN). Cluster heatmaps were generated using the pheatmap package, principal component analysis (PCA) and significant differential metabolite analysis were performed with the metaX package, partial least squares discriminant analysis (PLS-DA) was carried out using the ropls package with variable importance in projection (VIP) values calculated, and correlation analysis was conducted using Pearson’s correlation coefficient from the cor package. The final identification of significant differential metabolites was based on a *p*-value < 0.05 from the t-test, a fold change > 1.2, and a VIP > 1 from PLS-DA analysis. Kyoto Encyclopedia of Genes and Genomes (KEGG) pathway enrichment analysis was performed using hypergeometric tests, with a *p*-value < 0.05 indicating significant enrichment; metabolite set enrichment analysis was conducted using GSEA (version 4.1.0), and KEGG pathways with |NES| > 1 and a nominal *p*-value < 0.05 were considered significantly different between the two groups, with network diagrams constructed based on metabolite pathways to illustrate their interactions (Chen et al., 2022).

### 2.11 Statistical analysis

Data statistical analysis was carried out with SPSS 23.0. To compare means among multiple groups, one-way analysis of variance (ANOVA) was applied, and Tukey’s *post hoc* test was then used to pinpoint specific pairwise variations, the *p*-value less than 0.05 indicates a statistically significant difference, while a *p*-value less than 0.01 suggests a highly statistically significant difference. Additionally, for clarity in all tables, error bars represent ±SD (Standard Deviation) unless otherwise specifically stated. Data visualizations were created using GraphPad Prism 8 (Zhang X. et al., 2025).

## 3 Results

### 3.1 Effect of equine-derived *Lactobacillus* M11 on body weight and physical condition of pregnant female mice

During the entire experimental period, mice in all groups maintained a good mental state. No abnormalities in water intake and food consumption were observed macroscopically, and there were no signs of diarrhea or other diseases. There were no significant differences in fecal morphology and color among the groups, and no mortality occurred. The body weight data of the mice are presented in Table 1. On day 7, the M11-H group took the lead, with the BC group coming next. By day 14, the M11-H group peaked once again, followed by the BC group. Despite these variations, statistical analysis at all time points indicated no significant differences among the groups (Table 1).

**Table 1:** Body weight data of pregnant female mice across different groups during the experimental period.

Date	Groups				*F*-value	*p*-value
	BC (*n* = 6)	M11-L (*n* = 7)	M11-L (*n* = 6)	M11-H (*n* = 9)		
Day 0 (g)	36.63 ± 1.50	35.22 ± 1.81	34.77 ± 1.30	36.48 ± 1.66	2.206	0.113
Day 7 (g)	39.39 ± 2.01	37.87 ± 1.24	37.91 ± 1.24	39.59 ± 2.69	1.553	0.227
Day 14 (g)	48.86 ± 3.57	45.63 ± 3.51	46.17 ± 2.87	50.29 ± 7.01	1.55	0.227

### 3.2 Effect of equine-derived *Lactobacillus* M11 on offspring mice

The results of litter weight, number of offspring, and average birth weight of offspring rats across different groups are presented in Table 2. Compared to the M11-L group, both the M11-M and M11-H groups exhibited higher averages in terms of litter weight and number of offspring. The average birth weight of offspring rats in the M11-L, M11-M, and M11-H groups was higher than that in the BC group.

**Table 2:** Litter weight, number of offspring, and average birth weight of offspring in offspring mice from different groups.

Items	Groups				*F*-value	*p*-value
	BC (*n* = 6)	M11-L (*n* = 7)	M11-L (*n* = 6)	M11-H (*n* = 9)		
Litter weight (g)	27.31 ± 6.40	26.36 ± 1.96	27.33 ± 1.66	26.69 ± 5.76	0.072	0.974
Number of offspring (n)	16.50 ± 2.66	14.43 ± 1.81	15.17 ± 1.17	15.11 ± 4.34	0.538	0.661
Average birth weight of offspring rats (g)	1.66 ± 0.30	1.84 ± 0.12	1.81 ± 0.10	1.84 ± 0.37	0.656	0.587

### 3.3 Effect of equine-derived *Lactobacillus* M11 on serum biochemical indicators in pregnant female mice

The research results (Table 3) indicate that among multiple indicators, the *p*-values for albumin (ALB), alanine aminotransferase (ALT), and total cholesterol (TC) are less than 0.05, suggesting significant differences. Specifically, the mean albumin value in the M11-H group (40.30 ± 1.75 g/L) is higher than that in the other groups. The M11-L group has the highest mean alanine aminotransferase value (59.57 ± 10.34 U/L). The mean total cholesterol value in the M11-L group (2.90 ± 0.24 mmol/L) is significantly higher than that in the BC group (2.27 ± 0.20 mmol/L). No significant differences are observed for the other indicators among the groups.

**Table 3:** Serum biochemical indicators of pregnant female mice in different groups.

Items	Groups				*p*-value
	BC (*n* = 6)	M11-L (*n* = 7)	M11-L (*n* = 6)	M11-H (*n* = 9)	
Albumin (ALB, g/L)	38.17 ± 3.31^ab^	37.56 ± 1.14^b^	37.58 ± 1.91^b^	40.30 ± 1.75^a^	0.045*
Total Protein (TP, g/L)	70.98 ± 4.51	70.40 ± 2.94	70.87 ± 4.41	74.12 ± 3.02	0.18
Globulin (GLOB, g/L)	32.82 ± 1.24	32.83 ± 2.23	33.28 ± 2.84	33.54 ± 1.64	0.872
Total Bilirubin (TB)	1.32 ± 0.85	0.83 ± 0.76	1.22 ± 0.52	1.50 ± 0.48	0.381
Aspartate Aminotransferase (AST, U/L)	127.00 ± 16.42	146.71 ± 32.21	123.33 ± 40.09	124.33 ± 13.83	0.332
Alanine Aminotransferase (ALT, U/L)	52.17 ± 4.58^ab^	59.57 ± 10.34^a^	53.50 ± 8.04^ab^	47.78 ± 5.04^b^	0.032*
Alkaline Phosphatase (ALP, U/L)	71.17 ± 17.94	84.71 ± 13.85	72.83 ± 19.34	81.11 ± 15.94	0.405
Lipase (LPS, U/L)	61.33 ± 8.31	63.29 ± 6.58	67.83 ± 5.60	63.56 ± 3.50	0.308
Lactate Dehydrogenase (LDH, U/L)	647.67 ± 94.83	639.71 ± 122.05	588.83 ± 140.18	645.22 ± 126.43	0.807
Creatine Kinase (CK, U/L)	695.67 ± 266.19	689.71 ± 218.98	555.33 ± 184.80	799.44 ± 157.37	0.193
Creatinine (Crea, umol/L)	18.67 ± 4.26	17.94 ± 7.66	18.52 ± 5.61	20.15 ± 5.57	0.903
Uric Acid (UA, umol/L)	75.92 ± 26.56	66.54 ± 21.41	69.83 ± 51.57	60.42 ± 23.74	0.822
Blood Urea Nitrogen (BUN, mmol/L)	7.17 ± 0.83	8.10 ± 1.61	8.09 ± 2.80	7.71 ± 1.30	0.756
Total Cholesterol (TC, mmol/L)	2.27 ± 0.20^c^	2.90 ± 0.24^a^	2.77 ± 0.36^ab^	2.46 ± 0.37^bc^	0.004**
Triglyceride (TG, mmol/L)	1.51 ± 0.52	1.89 ± 0.47	1.88 ± 1.35	1.56 ± 0.51	0.703

Different lowercase superscript letters indicate a statistically significant difference with *p* < 0.05. “*” represents *p* < 0.05, “*” represents *p* < 0.01.

### 3.4 Impact of equine-derived *Lactobacillus* M11 on antioxidant indicators in pregnant female mice

The antioxidant results are presented in Table 4. Regarding the SOD indicator, the mean values of the M11-L and M11-L groups were higher than those of the BC and M11-H groups. For the GSH - PX indicator, the M11-L group had the highest mean value, while the M11-L group had the lowest. In terms of the MDA indicator, the M11-L group showed the lowest mean value. However, the F - values and *p* - values for each indicator indicated that there were no significant differences among the different groups.

**Table 4:** Antioxidant indicators (SOD, GSH-PX, T-AOC, MDA) in serum samples of pregnant KM mice across treatment groups.

Items	Groups				*F*-value	*p*-value
	BC (*n* = 6)	M11-L (*n* = 7)	M11-L (*n* = 6)	M11-H (*n* = 9)		
SOD (U/mL)	57.59 ± 3.70	62.40 ± 4.32	61.48 ± 4.44	58.24 ± 4.37	2.137	0.122
GSH-PX (U/mL)	290.94 ± 12.50	288.24 ± 29.25	311.54 ± 22.11	296.45 ± 21.60	1.337	0.286
T-AOC (U/mL)	6.02 ± 1.01	6.14 ± 0.91	5.97 ± 0.35	5.90 ± 0.72	0.127	0.943
MDA (nmol/mL)	4.23 ± 0.26	4.04 ± 0.51	3.91 ± 0.54	4.23 ± 0.50	0.759	0.528

### 3.5 Impact of equine-derived *Lactobacillus* M11 on immune indicators in pregnant female mice

The results of immune indicators are shown in Table 5. For the IL - 6 indicator, the M11-L group had the highest mean value, while the BC group had the lowest. Among the IgA indicators, the M11-L had the highest mean value across all groups, and the BC had the lowest. In terms of the IgG indicator, the BC had the highest mean value, and the M11-L group had the lowest. Regarding the IgM indicator, the M11-H group had the highest mean value, and the M11-L group had the lowest. However, the F - values and *p* - values for each indicator indicated that there were no significant differences in these immune indicators among the different concentration groups.

**Table 5:** Immune indicators (IL-6, IgA, IgG, IgM) in serum samples of pregnant KM mice receiving different concentrations of *Lactobacillus* M11.

Items	Groups				*F*-value	*p*-value
	BC (*n* = 6)	M11-L (*n* = 7)	M11-L (*n* = 6)	M11-H (*n* = 9)		
IL-6 (pg/mL)	76.12 ± 26.15^b^	109.16 ± 15.54^a^	96.17 ± 32.57^ab^	85.30 ± 26.19^ab^	2.084	0.129
IgA (μg/mL)	790.40 ± 228.56^b^	1032.34 ± 187.88^a^	828.77 ± 181.59^ab^	959.81 ± 209.33^ab^	2.041	0.135
IgG (g/L)	16.77 ± 6.09	13.28 ± 2.47	16.20 ± 2.62	15.32 ± 3.05	1.122	0.36
IgM (μg/mL)	1850.87 ± 339.71	1771.30 ± 421.36	2044.28 ± 581.83	2136.82 ± 316.80	1.246	0.315

Different lowercase superscript letters indicate a statistically significant difference with *p* < 0.05.

### 3.6 Localization of *Lactobacillus* M11 in the intestinal tract of mice

**Figure 1:**
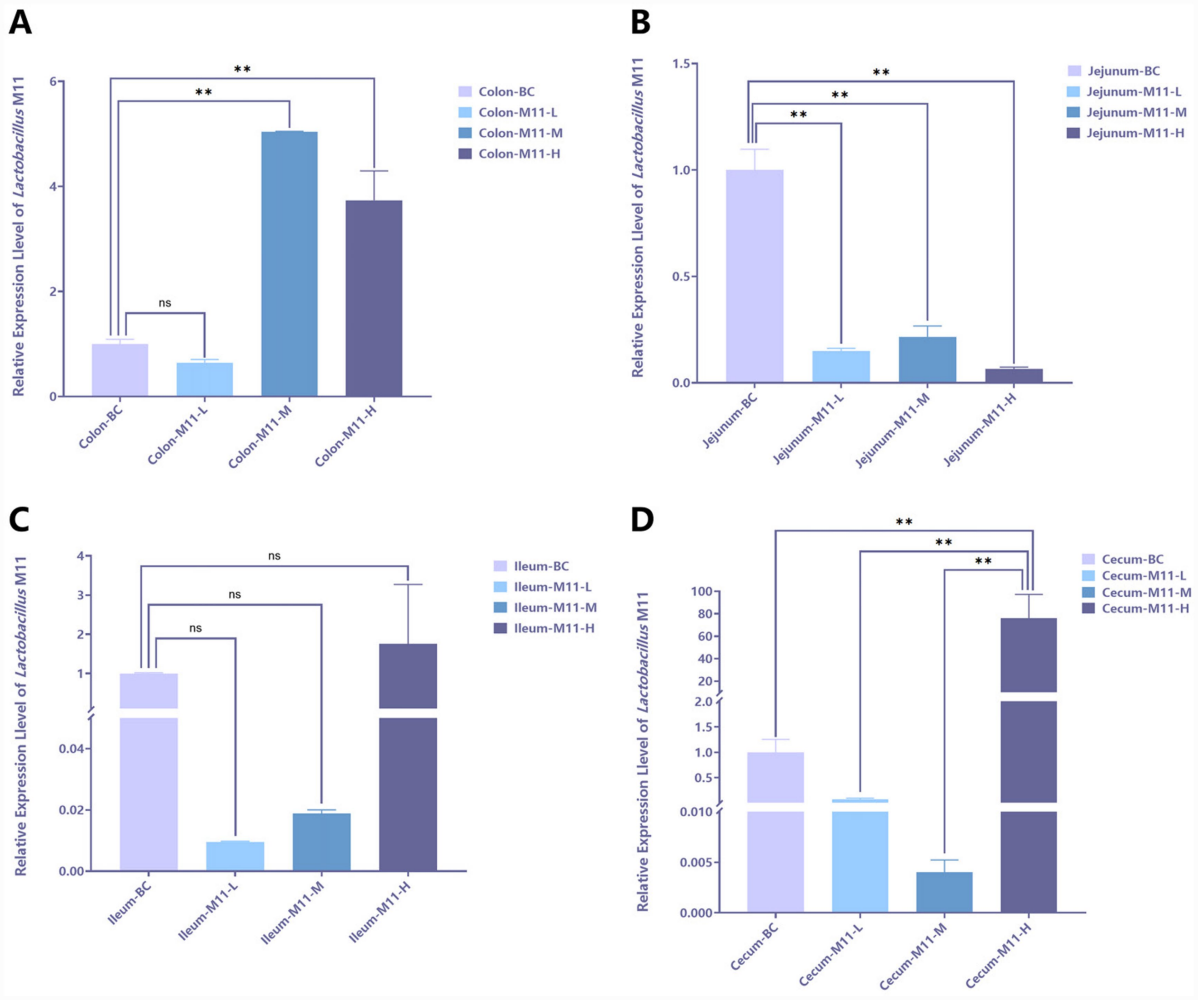
Localization of *Lactobacillus* M11 in the intestinal tract of mice. “**” *p* < 0.01, “ns” *p* > 0.05. **(A)** The relative expression level of *Lactobacillus* M11 in the colon. **(B)** The relative expression level of *Lactobacillus* M11 in the jejunum. **(C)** The relative expression level of *Lactobacillus* M11 in the ileum. **(D)** The relative expression level of *Lactobacillus* M11 in the cecum.

Figure 1 presents the results of the relative expression levels of *Lactobacillus* M11 in intestinal tissues across different groups. Figure 1A illustrates that, compared with the BC group, *Lactobacillus* M11 exhibits higher expression levels in the colon of the M11-M and M11-H groups. Figure 1B demonstrates that, in the jejunum, the BC group shows the highest expression level of *Lactobacillus* M11. Figure 1C indicates that there are no significant differences in the expression levels of *Lactobacillus* M11 in the ileum among all groups. Figure 1D reveals that *Lactobacillus* M11 has the highest expression level in the cecum, and the expression level in the M11-H group is extremely significantly higher than those in the other groups. Therefore, for subsequent sequencing, we selected the cecal contents from the BC and M11-H groups for comparison.

### 3.7 Impact of equine-derived *Lactobacillus* M11 on metagenomic in pregnant female mice

#### 3.7.1 PCoA and NMDS analyses of the microbiome groups

**Figure 2:**
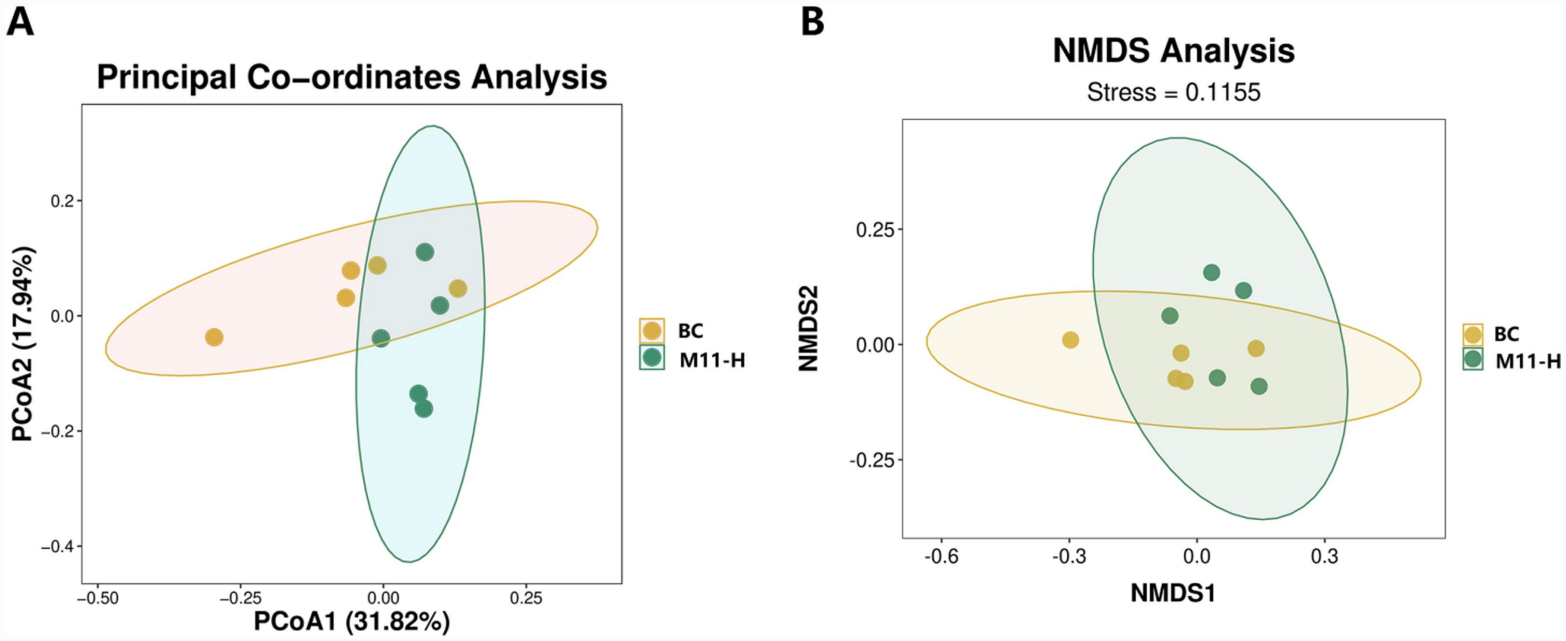
PCoA and NMDS analyses of the microbiome groups. **(A)** PCoA analysis: In the results, different colors represent different groups. The closer the distance between samples, the more similar the microbial composition structures are among the samples, and the smaller the differences. **(B)** NMDS analysis: The points in the graph represent samples, and samples of different colors belong to different groups. The distance between points indicates the degree of difference between samples. The stress is employed to evaluate the performance of the NMDS analysis. It is generally considered that when stress < 0.2, the two-dimensional point plot of NMDS can be used for representation, and the graph has certain explanatory significance.

Principal coordinate analysis (PCoA) was conducted based on the Bray-Curtis distance. Through PCoA (Figure 2A), differences among groups could be observed. Nonmetric multidimensional scaling (NMDS) analysis (Figure 2B) revealed that with a stress value < 0.2, the two-dimensional scatter plot of NMDS could be used to demonstrate the differences among groups.

#### 3.7.2 MetagenomeSeq differential analysis

**Figure 3:**
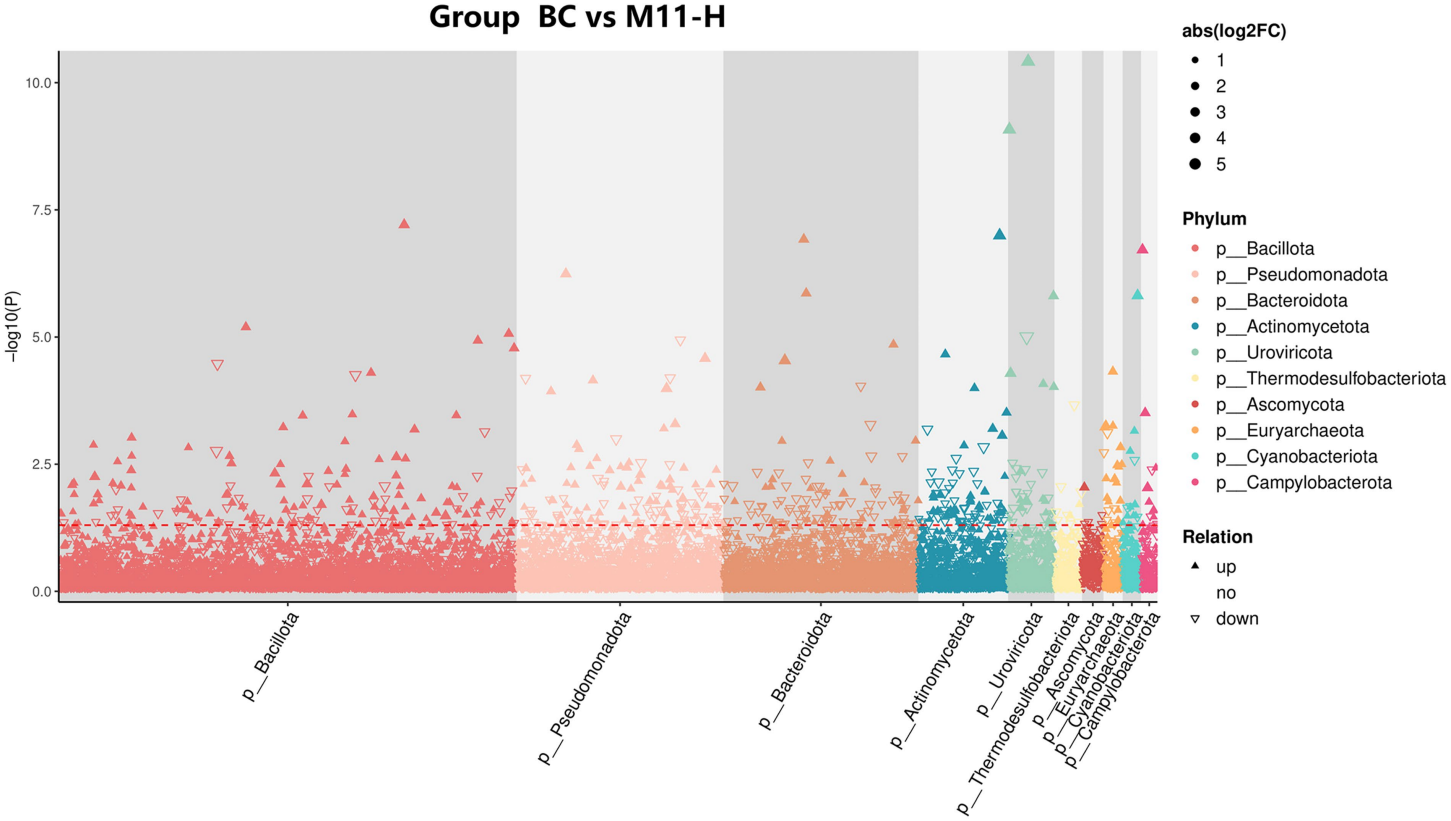
MetagenomeSeq differential analysis. The points in the graph represent species at the species level (i.e., the taxonomic rank of species). Different colors denote species at the phylum level. The vertical axis represents the -log10 (*p*-value) value, with a higher position on the Y-axis indicating greater significance of the difference. The dashed line demarcates significant differences; species above the dashed line are considered significant. The upward-pointing triangles signify up-regulated differences (i.e., increased abundance), while the downward-pointing triangles indicate down-regulated differences (i.e., decreased abundance). The dots represent species with no significant differences.

MetagenomeSeq analysis is predominantly employed to compare the abundance differences of microbial species at various taxonomic levels between two sets of samples (Jagadesan and Guda, 2025). Using |logFC| > 1 and a *p*-value < 0.05 as the significance thresholds for differential screening, it identifies species with significant differences between the two sample groups. Based on the results of the differential analysis, the findings are visualized using a Manhattan plot (Figure 3). Species such as *Bacillota*, *Pseudomonadota*, *Bacteroidota*, and *Actinomycetota* exhibit differences between the two groups. These differences are at the phylum level, and details of differences at the species level can be found in Supplementary Table 6.

#### 3.7.3 Reporter score-based pathway enrichment analysis

**Figure 4:**
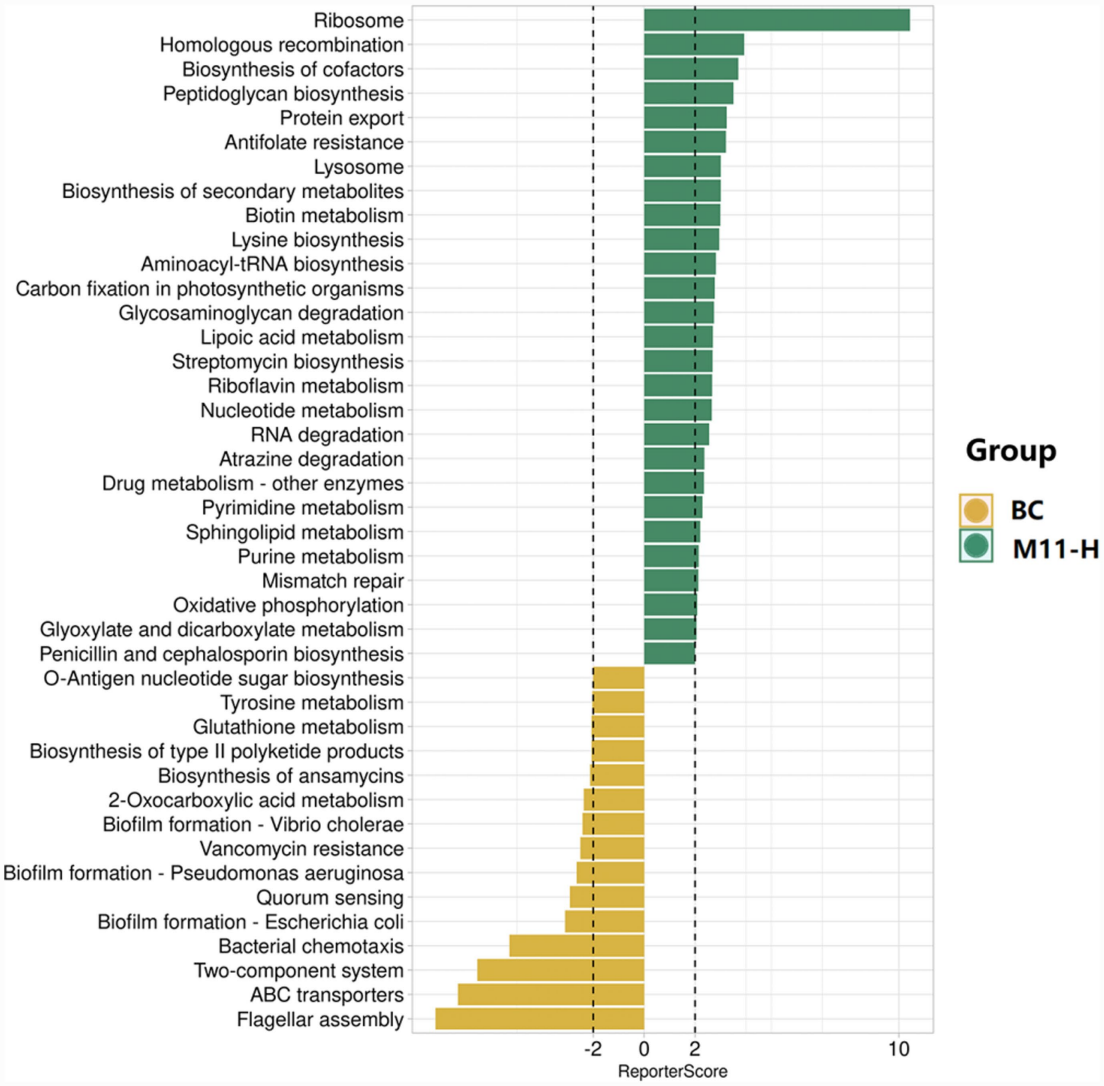
Reporter score enrichment analysis. In the figure, a taller bar indicates a larger |Reporter Score| value and a higher degree of enrichment. The colors of the bars represent the groups in which the pathways are significantly enriched. Under the “directed” mode, the positive or negative value of the reporter score signifies an increasing or decreasing trend of the pathway, respectively. Under the “mixed” mode, the sign of the reporter score does not indicate the trend of the pathway; instead, a larger reporter score value corresponds to a higher degree of enrichment.

Pathways with |Reporter Score| greater than 2 were selected to plot a histogram (Figure 4). The results indicated that the M11-H group was significantly enriched in pathways such as “O-Antigen nucleotide sugar biosynthesis”, “Tyrosine metabolism”, and “Glutathione metabolism.”

### 3.8 Impact of equine-derived *Lactobacillus* M11 on metabolism in pregnant female mice

#### 3.8.1 PLS-DA analysis among metabolite groups

**Figure 5:**
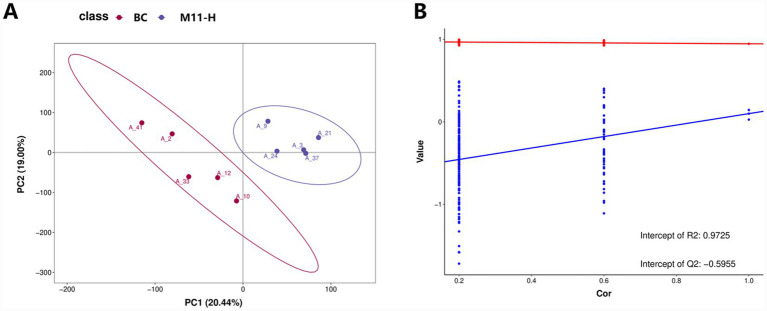
PLS-DA analysis: separation of metabolite groups in pregnant female mice treated with equine-derived *Lactobacillus*. **(A)** PLS-DA score plot: the *x*-axis represents the first principal component PC1, and the *y*-axis represents the second principal component PC2. Each point in the plot represents a sample, with different groups displayed in distinct colors. The relative positions of the points reflect the degree of dispersion among the samples. Samples that are closer in relative distance indicate more similar expression patterns. **(B)** Permutation test plot: the grouping labels of each sample are randomly shuffled, followed by modeling and prediction. Each modeling process corresponds to a set of R^2^ and Q^2^ values. Based on the Q^2^ and R^2^ values obtained from 200 rounds of shuffling and modeling, their regression lines can be derived. In the plot, the R^2^ regression line is shown in red, and the Q^2^ regression line is shown in blue. When the *x*-axis is within the range of [0, 1], and the R^2^ regression line lies above the Q^2^ regression line while the *y*-intercept of the Q^2^ regression line is less than 0, it indicates that the model is not overfitted.

According to the PLS-DA analysis (Figure 5A), samples from the BC and M11-H groups showed a certain degree of separation in the principal component space. This indicates that *Lactobacillus* M11 has differential impacts on the overall metabolome of the cecal contents of pregnant female mice. The permutation test plot (Figure 5B) demonstrates that the model is not overfitted.

#### 3.8.2 Analysis of differential metabolites

**Figure 6:**
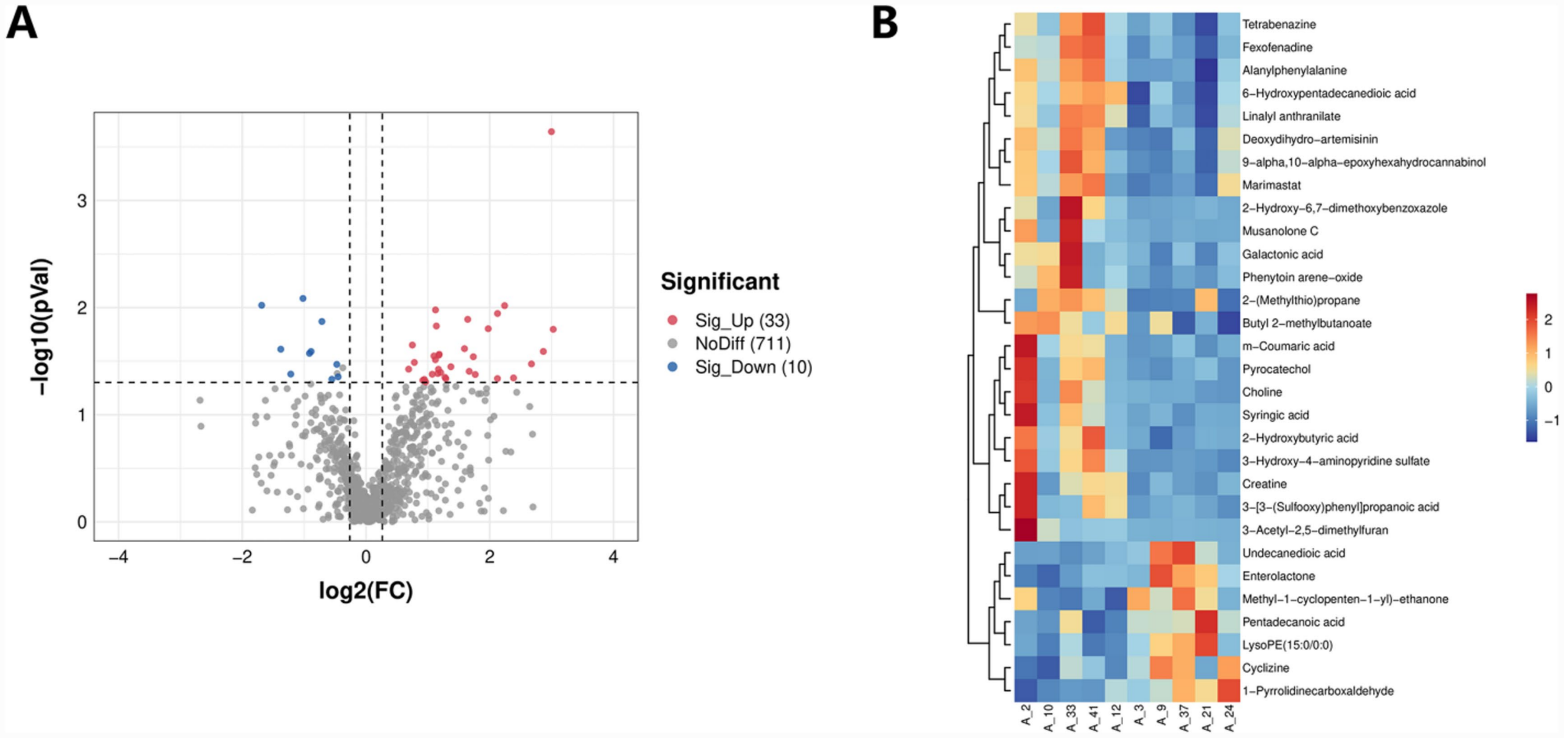
Volcano and heatmap analysis of differential metabolites in pregnant female mice. **(A)** Volcano plot. Using log_2_ (FC) as the *x*-axis and -log_10_ (*p*-value) as the *y*-axis, a volcano plot is generated for all metabolites analyzed in the differential expression study. In this plot, red points represent significantly upregulated differential metabolites, blue points denote significantly downregulated differential metabolites, and gray points indicate non-significantly differential metabolites. **(B)** Heatmap of differential metabolites: In this heatmap, the *x*-axis represents samples, while the *y*-axis displays the selected differentially expressed metabolites. By default, the top 30 differential metabolites are visualized in the heatmap. Different colors indicate varying relative abundances of metabolites: red signifies relatively high abundance, and blue denotes relatively low abundance. The more intensely red or blue a color appears, the higher or lower, respectively, the relative abundance of the metabolite is across different groups.

The overall distribution of differential metabolites can be understood by creating a volcano plot (Figure 6A). Among the samples from the BC and M11-H groups, the majority of metabolites (711) showed no significant differences in expression. However, 33 metabolites were significantly upregulated, and 10 metabolites were significantly downregulated. The heatmap of differential metabolites (Figure 6B) displays the top 30 differential metabolites, which encompass various types, including benzodiazepines (e.g., Tetrabenazine), components related to antifungal and lipid-regulating drugs, amino acids and their derivatives (e.g., Alanylphenylalanine, Choline, Syringic acid), organic acids (e.g., Galactonic acid, m-Coumaric acid), terpenoids, and oxygen-containing heterocyclic compounds.

#### 3.8.3 KEGG enrichment analysis

**Figure 7:**
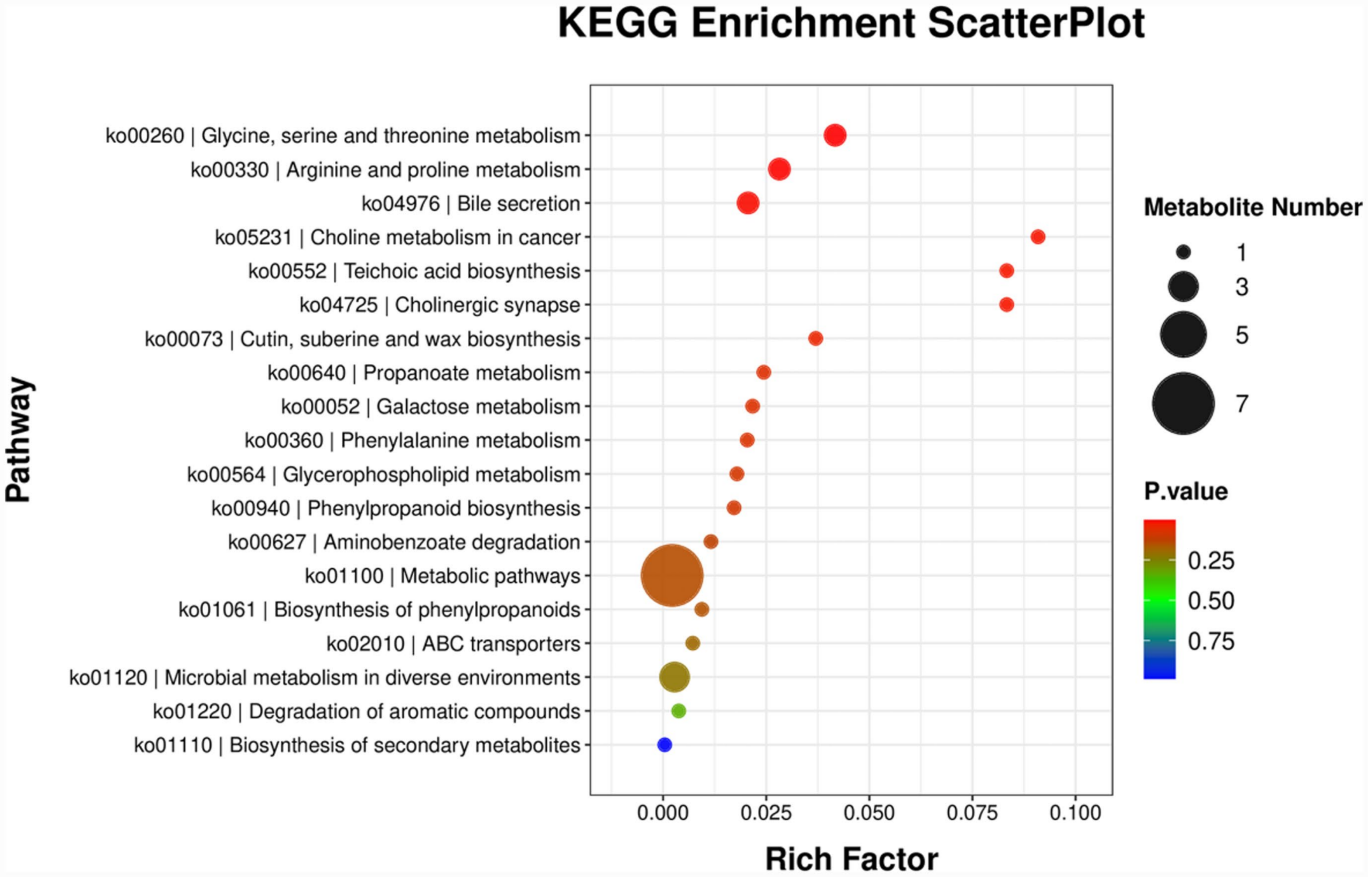
KEGG enrichment analysis of metabolic pathways in pregnant female mice. The RichFactor represents the ratio of the number of differential metabolites located in a particular pathway to the total number of metabolites contained in that pathway. A larger RichFactor value indicates a greater degree of enrichment in that pathway. In the scatter plot, the size of each point represents the number of differential metabolites on the corresponding pathway, while the color of the point corresponds to the *p*-value from the enrichment analysis, reflecting the significance of the enrichment.

The top 20 pathways with the smallest *p*-values were selected and presented in a bubble plot (Figure 7). These pathways were significantly enriched in “Glycine, serine and threonine metabolism,” “Arginine and proline metabolism,” “Bile secretion,” “Metabolic pathways,” “Cutin, suberine and wax biosynthesis,” and other pathways.

### 3.9 Integrated analysis of microorganisms and metabolism

**Figure 8:**
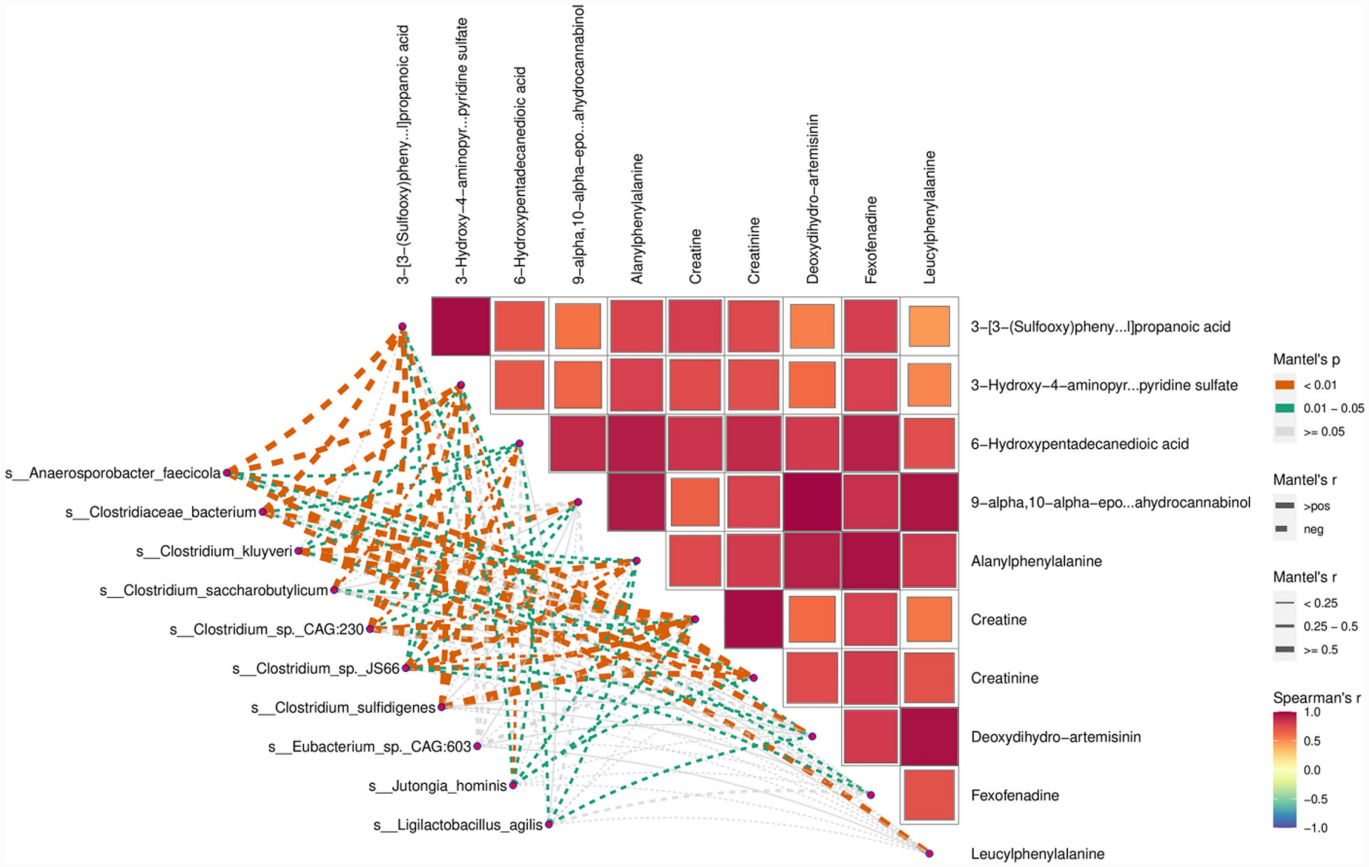
Mantel test analysis. In the heatmap on the right side, the color of each square represents the strength of the correlation between metabolomes. The redder the color, the closer the correlation coefficient is to 1, indicating a stronger positive correlation; conversely, the bluer the color, the closer the correlation coefficient is to −1, indicating a stronger negative correlation. The network diagram in the bottom-left corner presents the results of the correlation analysis between 10 differentially abundant species and 10 differentially abundant metabolites. The colors represent *p*-values, while the thickness of the lines represents *r*-values. A thicker line indicates a stronger correlation.

Integrated analysis was conducted on the differential microbial species and differential metabolites between the BC and M11-H groups. Figure 8 demonstrates that the Bacillota (specifically, *s_Clostridiaceae_bacterium*) exhibits a significant positive correlation with metabolite modules, such as 3-Hydroxy-4-aminopyridine sulfate and 3-[3-(Sulfooxy)phenyl]propanoic acid.

## 4 Discussion

This study systematically explored the effects of equine-derived *Lactobacillus* M11 on the reproductive performance and metabolic profile of pregnant KM mice, aiming to evaluate the potential of M11 as a safe alternative in antibiotic-free farming and to fill a critical gap in the field of probiotic-mediated reproductive support. The following is a detailed discussion of the results.

### 4.1 Physiological and biochemical effects

Serum biochemical indicators revealed group-specific differences. Albumin (ALB), synthesized by the liver, is the most abundant and functionally diverse protein in plasma. It prevents edema caused by fluid exudation by maintaining plasma colloid osmotic pressure, ensuring water balance between the intra- and extravascular spaces (Matsui et al., 2025; Zhang Y. et al., 2025). It also acts as a “carrier” for various substances, binding and transporting fatty acids, bilirubin, hormones, and drugs to ensure their stable delivery to target tissues (Bodega et al., 2002; Hillmer et al., 2023). Additionally, it participates in maintaining blood pH stability, exhibits antioxidant properties, and protects cells from free radical damage, playing a crucial supportive role in physiological processes such as metabolism, immunity, and pregnancy (Aravind and Krishnan, 2016; Gremese et al., 2023). The elevated ALB levels in the M11-H group suggest improved nutritional status or enhanced hepatic protein synthesis. Alanine aminotransferase (ALT), primarily found in hepatocytes, is involved in amino acid metabolism. When hepatocytes are damaged, ALT is released into the bloodstream in large quantities, leading to elevated serum levels. It serves as an important indicator for assessing liver health, diagnosing liver diseases, and monitoring disease progression (Wedemeyer et al., 2010). Total cholesterol (TC) represents the sum of cholesterol in all lipoproteins in the blood and plays physiological roles in forming cell membranes, synthesizing bile acids, and hormones. Its levels are closely associated with the risk of cardiovascular diseases and serve as a key indicator for evaluating lipid metabolism and cardiovascular health (Liu et al., 2023). The transient increases in ALT and TC levels in the M11-L group align with adaptive changes in hepatic lipid metabolism. Notably, the elevated TC levels in the M11-L group are consistent with previous findings where specific probiotics enhanced lipid absorption. Although antioxidant (SOD, GSH-PX) and immune (IL-6, IgA/IgG/IgM) indicators showed no statistical differences, they exhibited dose-dependent trends (e.g., elevated SOD in the M11-L/M11-M groups and reduced MDA in the M11-M group). SOD, a potent antioxidant enzyme, converts superoxide anion radicals into harmless oxygen and hydrogen peroxide, reducing cellular damage caused by free radicals, delaying aging, preventing diseases, protecting cell membrane integrity, enhancing immunity, and improving skin condition (McCord and Edeas, 2005; Adisti et al., 2025). MDA, the end product of lipid peroxidation, disrupts cell membrane structure and function, leading to cellular damage. Its levels reflect the extent of lipid peroxidation in the body and indirectly measure cellular damage and aging status (Tsikas et al., 2016; Tsikas, 2023). The results of this trial suggest subtle regulatory effects of *Lactobacillus* M11 on oxidative stress. However, these trends require validation of dose–response relationships through studies with larger sample sizes.

### 4.2 Microbial regulation and reproductive impacts

Both PCoA and NMDS analyses demonstrated significant separation in the microbial community space between the BC group and the M11-H group, with an NMDS stress value < 0.2 validating the credibility of the two-dimensional space (Wachsmannová et al., 2025). This separation was not merely a change in species abundance but reflected the reconfiguration of microbial community niches. Specifically, the abundance differences in core phyla such as *Bacillota* and *Pseudomonadota* represented an ecological marker of the transition of the microbial community from random fluctuations to functionally directed states. For instance, the significant enrichment of *Bacillota* may enhance short-chain fatty acid synthesis capacity, thereby improving intestinal barrier function and providing a more stable nutritional microenvironment for fetal development (Qin et al., 2025). Conversely, the differences in *Pseudomonadota* may involve the activation of immune regulatory pathways, such as promoting immune tolerance through the secretion of extracellular polysaccharides and reducing the negative reproductive impacts of inflammatory responses during pregnancy (Selle et al., 2022).

The phylum-level differences revealed by MetagenomeSeq differential analysis did not exist in isolation but formed causal chains with the enrichment of specific metabolic pathways (Weiss et al., 2017). Taking Bacillota as an example, its significant enrichment in the M11-H group was directly associated with the activation of the “O-antigen nucleotide sugar biosynthesis” pathway (Reporter Score > 2). This pathway participates in intestinal mucosal immune regulation by synthesizing glycoconjugates, potentially enhancing maternal-fetal immune protection by promoting IgA secretion (Cerutti, 2008; Duerr et al., 2009). Additionally, the enrichment of the “tyrosine metabolism” pathway may influence maternal stress responses by regulating neurotransmitter synthesis, indirectly improving the reproductive environment. Notably, the significant enrichment of the “glutathione metabolism” pathway (Reporter Score > 2) not only reflected the antioxidant support provided by Equine-derived *Lactobacillus* but also likely influenced follicular development and embryo implantation by regulating redox balance, establishing a direct functional link from intestinal microbiota to the reproductive system (Bansal and Simon, 2018).

The metabolic pathway changes revealed by Reporter Score pathway enrichment analysis hold clear reproductive biological significance. The activation of the “O-antigen nucleotide sugar biosynthesis” pathway not only involves intestinal immune regulation but also suggests that the synthesized glycoconjugates may influence the reproductive tract microbiota via the bloodstream, forming a long-distance regulatory “gut-reproductive axis” (Ashonibare et al., 2024). The enrichment of the “tyrosine metabolism” pathway may affect uterine contractility and angiogenesis by regulating catecholamine synthesis, providing optimal blood perfusion for fetal development (Fernstrom and Fernstrom, 2007). Meanwhile, the strengthening of the “glutathione metabolism” pathway is directly related to the antioxidant capacity of follicular fluid, potentially improving oocyte quality by reducing oxidative stress damage (Kurdi et al., 2025).

### 4.3 Metabolic regulation and reproductive impacts

Partial least squares discriminant analysis (PLS-DA) revealed a clear separation between the blank control group (BC) and the M11-H group in the cecal metabolome space, confirming that *Lactobacillus* M11 induced a unique metabolic transition. This separation is consistent with established mechanisms of probiotic action, whereby the gut microbiota regulates host metabolism through metabolite-mediated signaling (Falcinelli et al., 2018; Dong et al., 2025; Li et al., 2025). KEGG enrichment analysis identified “glycine, serine, and threonine metabolism” and “arginine and proline metabolism” as the most significantly enriched pathways. These pathways hold significant biological importance: glycine supports collagen synthesis in placental blood vessels, while arginine-derived nitrogen oxides regulate uterine blood flow—both are essential for fetal development (Bodis et al., 2022; de Paz-Lugo et al., 2023; Kurhaluk and Tkaczenko, 2025). The upregulation of these pathways indicates that *Lactobacillus* M11 enhances metabolic support during pregnancy, particularly in amino acid turnover and tissue remodeling.

Volcano plots and heatmap analyses further characterized the differential metabolites, revealing 33 upregulated and 10 downregulated metabolites in the M11-H group compared to the BC group. Key metabolites included amino acid derivatives (e.g., alanyl-phenylalanine, choline) and polyphenolic substances (e.g., syringic acid). Choline, a precursor to phosphatidylcholine, is crucial for fetal membrane integrity and lipid metabolism (Zeisel, 2006; Jaiswal et al., 2023). The antioxidant properties of syringic acid may alleviate pregnancy-related oxidative stress (Srinivasulu et al., 2018; Demir, 2024). Correlation network analysis highlighted hub metabolites such as sinapic acid and syringic acid, emphasizing their central role in metabolic interactions—a finding of significant importance for identifying biomarkers of probiotic efficacy.

### 4.4 Integrated analysis of microbial and metabolic regulation

This study, through an integrated analysis of microorganisms and metabolites, reveals the collaborative regulatory network between the intestinal microbiota and metabolic profiles in pregnant mice intervened by Equine-derived *Lactobacillus* M11. The core finding—the significant positive correlation between the specific bacterium *s_Clostridiaceae_bacterium* within the phylum Bacillota and metabolites such as 3-hydroxy-4-aminopyridine sulfate and 3-[3-(sulfooxy)phenyl]propanoic acid—holds profound reproductive biological implications.

The positive correlation between *s_Clostridiaceae_bacterium*, a core species within the phylum Bacillota, and specific metabolites is not coincidental but rather reflects the directional reconfiguration of microbial community functional modules. The phylum Bacillota commonly participates in energy metabolism and immune regulation in the gut through the synthesis of short-chain fatty acids (e.g., butyric acid) (Candeliere et al., 2023). The enrichment of *s_Clostridiaceae_bacterium* may specifically activate amino acid metabolic pathways. As a nitrogen-containing heterocyclic compound, 3-hydroxy-4-aminopyridine sulfate may be an extended product of arginine/proline metabolism, and its accumulation may improve placental blood flow by enhancing nitric oxide synthesis (Lau et al., 2000). Meanwhile, as a phenylpropanoid derivative, the sulfonated structure of 3-[3-(sulfooxy)phenyl]propanoic acid may reduce oxidative stress-induced damage to oocytes by enhancing antioxidant capacity (Grace and Logan, 2000). This “species-metabolite” association essentially represents a precise matching between microbial functional modules and host metabolic demands.

The functional analysis of 3-hydroxy-4-aminopyridine sulfate and 3-[3-(sulfooxy)phenyl]propanoic acid necessitates a reproductive physiological perspective. The former may regulate maternal-fetal signaling by participating in neurotransmitter synthesis (e.g., glutamate metabolism), with its sulfate group potentially enhancing metabolite stability and bioavailability to promote fetal neural development (Sun et al., 2023). The latter, as a sulfur-containing antioxidant, may protect theca cells by scavenging reactive oxygen species (ROS), reducing the risk of premature ovarian failure, while its phenylpropanoid backbone may improve endometrial receptivity by activating estrogen receptors (Shi et al., 2023). This dual role of metabolites—serving as both energy substrates and signaling molecules—forms a complete logical chain from metabolic support to developmental programming.

The enrichment of *s_Clostridiaceae_bacterium* may regulate intestinal barrier function by producing specific metabolites (e.g., short-chain fatty acids), reducing chronic inflammation triggered by endotoxin translocation into the bloodstream, thereby improving the reproductive environment. Meanwhile, the accumulation of metabolites may reach reproductive organs such as the ovaries and uterus via the bloodstream, forming a cascading regulatory network of “intestinal microbiota-metabolites-reproductive organs” (Fu et al., 2025). This global integrative effect is not only manifested at the level of individual pathways but also forms multidimensional reproductive support through the cross-talk of metabolic networks.

## 5 Conclusion

This study demonstrates that equine-derived *Lactobacillus* M11 enhances the reproductive performance of pregnant KM mice through multidimensional regulation encompassing physiological, biochemical, microbial, and metabolic pathways. These findings position M11 as a promising probiotic candidate for antibiotic-free agriculture, offering mechanistic insights, providing a scientific foundation for sustainable agriculture, and facilitating the optimization of livestock reproductive efficiency. However, due to the short intervention period, further research is required to determine the optimal dosage and long-term effects.

## Data Availability

The original contributions presented in the study are included in the article/Supplementary material, further inquiries can be directed to the corresponding author.
