# Supplementary material for: Effect of equine-derived Lactobacillus M11 on the reproductive performance of KM pregnant female mice
Source: Front Microbiol. 2026 Feb 2;17:1741988. doi: 10.3389/fmicb.2026.1741988 (PMC12907403; doi:10.3389/fmicb.2026.1741988)
Supplement: Supplementary file 1 [file Table_1.docx]

Supplementary Material

**Supplementary Table 1.** Feed intake of mice.

| **Group** | **Identification Number** | **Feed Intake/g** | | |
| --- | --- | --- | --- | --- |
|  |  | **Day 0** | **Day 7** | **Day 14** |
| BC | A2 | 4.85 | 8.40 | 6.81 |
| BC | A10 | 7.41 | 8.33 | 8.25 |
| BC | A12 | 7.33 | 8.31 | 8.23 |
| BC | A33 | 7.46 | 7.32 | 8.67 |
| BC | A35 | 7.25 | 7.36 | 8.63 |
| BC | A41 | 6.62 | 6.16 | 6.74 |
| M9 | A7 | 6.34 | 5.45 | 6.43 |
| M9 | A23 | 6.31 | 5.41 | 6.42 |
| M9 | A9 | 8.87 | 8.92 | 9.74 |
| M9 | A16 | 8.89 | 8.89 | 9.87 |
| M9 | A21 | 8.95 | 7.93 | 8.81 |
| M9 | A24 | 8.98 | 7.95 | 8.84 |
| M9 | A3 | 7.19 | 6.72 | 7.12 |
| M9 | A37 | 7.22 | 6.88 | 7.25 |
| M9 | A29 | 5.05 | 6.87 | 7.71 |
| M7 | A22 | 6.19 | 6.64 | 7.04 |
| M7 | A13 | 6.42 | 7.42 | 7.65 |
| M7 | A27 | 6.46 | 7.45 | 7.67 |
| M7 | A8 | 5.79 | 6.56 | 6.31 |
| M7 | A20 | 5.82 | 6.57 | 6.32 |
| M7 | A43 | 6.67 | 6.36 | 6.29 |
| M7 | A46 | 6.64 | 6.38 | 6.31 |
| M8 | A19 | 7.24 | 6.73 | 6.24 |
| M8 | A25 | 6.41 | 7.74 | 6.06 |
| M8 | A14 | 5.87 | 6.92 | 6.85 |
| M8 | A17 | 5.82 | 6.91 | 6.87 |
| M8 | A26 | 5.27 | 6.02 | 6.94 |
| M8 | A45 | 5.67 | 5.83 | 7.50 |

**Supplementary Table 2.** Weights of mice organs.

| **Group** | **Identification Number** | **Organs Weight/g** | | |  |
| --- | --- | --- | --- | --- | --- |
|  |  | **Liver** | **Both kidneys** | **Spleen** | **Intestine** |
| BC | A2 | 3.61 | 0.59 | 0.24 | 5.31 |
| BC | A10 | 3.27 | 0.63 | 0.23 | 3.81 |
| BC | A12 | 2.76 | 0.5 | 0.19 | 3.90 |
| BC | A33 | 3.94 | 0.65 | 0.17 | 3.27 |
| BC | A35 | 4.19 | 0.62 | 0.3 | 6.12 |
| BC | A41 | 3.21 | 0.55 | 0.18 | 3.72 |
| M9 | A7 | 2.68 | 0.48 | 0.15 | 4.03 |
| M9 | A23 | 2.69 | 0.59 | 0.21 | 3.72 |
| M9 | A9 | 3.43 | 0.68 | 0.29 | 5.36 |
| M9 | A16 | 3.22 | 0.58 | 0.22 | 4.59 |
| M9 | A21 | 3.19 | 0.54 | 0.24 | 4.67 |
| M9 | A24 | 2.74 | 0.54 | 0.16 | 3.51 |
| M9 | A3 | 3.36 | 0.58 | 0.19 | 5.38 |
| M9 | A37 | 2.73 | 0.59 | 0.16 | 4.07 |
| M9 | A29 | 2.73 | 0.57 | 0.13 | 4.33 |
| M7 | A22 | 3.18 | 0.56 | 0.21 | 4.19 |
| M7 | A13 | 2.95 | 0.56 | 0.14 | 4.49 |
| M7 | A27 | 3.12 | 0.57 | 0.23 | 4.52 |
| M7 | A8 | 3.07 | 0.53 | 0.11 | 4.27 |
| M7 | A20 | 3.2 | 0.56 | 0.19 | 4.48 |
| M7 | A43 | 3.78 | 0.48 | 0.16 | 5.51 |
| M7 | A46 | 2.95 | 0.45 | 0.19 | 5.00 |
| M8 | A19 | 3.16 | 0.51 | 0.17 | 4.61 |
| M8 | A25 | 3.86 | 0.62 | 0.13 | 5.5 |
| M8 | A14 | 3.15 | 0.52 | 0.13 | 4.30 |
| M8 | A17 | 2.96 | 0.49 | 0.12 | 3.52 |
| M8 | A26 | 4.03 | 0.56 | 0.25 | 5.5 |
| M8 | A45 | 4.13 | 0.64 | 0.21 | 5.19 |

**Supplementary Table 3.** Reverse transcription system.

| **Reagents** | **Utilization Rate** | **Final Concentration** |
| --- | --- | --- |
| 5× PrimeScript RT Master Mix（Perfect Real Time） | 4 μL | 2× |
| Total RNA | （1000/C_RNA_）μL |  |
| RNase Free dH2O | up to 20 μL |  |

**Supplementary Table 4.** Primer information.

| **Gene Name** | **Primer Information** |
| --- | --- |
| *Lactobacillus* M11 | F:5’-GCTGGCAACTGACAACAAGG-3’  R:5’-ACAAAGTGACAGGTGGTGCA-3’ |
| *β-actin* | F:5’-GAGACCTTCAACACCCCAGC-3’  R:5’-ATGTCACGCACGATTTCCC-3’ |

**Supplementary Table 5.** Real-time fluorescence quantitative PCR reaction system.

| **Reagents** | **Utilization Rate** |
| --- | --- |
| TB Green Premix Ex Taq II （Tli RNaseH Plus） 2X | 5 μL |
| Forward and reverse primers | 0.8 μL |
| cDNA template + RNase-free water | 2 μL |
| Total reaction volume | 2.2 μL |

**Supplementary Table 6.** Results of species-level differential analysis using MetagenomeSeq (first 50 rows).

| **Species** | **logFC** | **se** | ***p-*values** | **adj *p-*values** |
| --- | --- | --- | --- | --- |
| *s__Podoviridae_sp._ctbd591* | 5.58 | 0.84 | 0.00 | 0.00 |
| *s__Bacillus_phage_vB_BceM-HSE3* | 5.09 | 0.83 | 0.00 | 0.00 |
| *s__Pamirivirus_faecium* | -5.07 | 1.15 | 0.00 | 0.01 |
| *s__Streptomyces_sp._CBMA29* | 4.78 | 0.90 | 0.00 | 0.00 |
| *s__Croceivirga_thetidis* | 4.24 | 1.01 | 0.00 | 0.02 |
| *s__Pseudanabaena_sp._PCC_7367* | 3.97 | 0.83 | 0.00 | 0.00 |
| *s__Candidatus_Desantisbacteria_bacterium_CG07_land_8_20_14_0_80_39_15* | 3.84 | 0.79 | 0.00 | 0.00 |
| *s__Desulfuribacillus_stibiiarsenatis* | -3.82 | 1.22 | 0.00 | 0.39 |
| *s__Becedseptimavirus_BCD7* | 3.80 | 0.94 | 0.00 | 0.03 |
| *s__Mycoplasma_mycoides* | 3.77 | 1.01 | 0.00 | 0.08 |
| *s__Arcobacter_sp._F2176* | 3.74 | 0.72 | 0.00 | 0.00 |
| *s__Pseudoxanthomonas_suwonensis* | 3.69 | 0.95 | 0.00 | 0.05 |
| *s__Thiococcus_pfennigii* | 3.56 | 0.85 | 0.00 | 0.02 |
| *s__Pseudoalteromonas_sp._MMG012* | -3.48 | 1.56 | 0.03 | 1.00 |
| *s__Rummeliibacillus_sp._POC4* | 3.46 | 1.62 | 0.03 | 1.00 |
| *s__Chitinolyticbacter_albus* | 3.46 | 0.69 | 0.00 | 0.00 |
| *s__Dialister_sp._CAG:588* | -3.40 | 0.82 | 0.00 | 0.02 |
| *s__Peptococcaceae_bacterium_SCADC1_2_3* | 3.39 | 0.63 | 0.00 | 0.00 |
| *s__Desulfurella_unclassified* | 3.39 | 1.30 | 0.01 | 0.79 |
| *s__Phage_Phass-1* | 3.34 | 1.81 | 0.06 | 1.00 |
| *s__Ornithinibacillus_massiliensis* | -3.31 | 0.82 | 0.00 | 0.03 |
| *s__Parenemella_sanctibonifatiensis* | -3.29 | 1.03 | 0.00 | 0.34 |
| *s__Marine_Group_I_thaumarchaeote_SCGC_AAA799-O18* | 3.24 | 0.69 | 0.00 | 0.00 |
| *s__Lactobacillus_selangorensis* | 3.16 | 1.19 | 0.01 | 0.78 |
| *s__Alkalihalobacterium_chitinilyticum* | 3.16 | 1.62 | 0.05 | 1.00 |
| *s__Crossiella_sp._SN42* | 3.15 | 1.41 | 0.03 | 1.00 |
| *s__Paenibacillus_sp._LC231* | -3.14 | 1.19 | 0.01 | 0.78 |
| *s__Candidatus_Portnoybacteria_bacterium_RIFCSPHIGHO2_01_FULL_40_12b* | 3.11 | 1.06 | 0.00 | 0.54 |
| *s__Flavobacteriia_bacterium_40-80* | 3.09 | 0.58 | 0.00 | 0.00 |
| *s__uncultured_Mediterranean_phage_uvDeep-CGR2-KM18-C269* | 3.07 | 0.64 | 0.00 | 0.00 |
| *s__Aquibacillus_salsiterrae* | 3.07 | 1.11 | 0.01 | 0.66 |
| *s__Campylobacter_sp._2018MI10* | 3.05 | 0.85 | 0.00 | 0.12 |
| *s__Candidatus_Peregrinibacteria_bacterium_CG11_big_fil_rev_8_21_14_0_20_49_14* | 3.04 | 0.65 | 0.00 | 0.00 |
| *s__Bifidobacterium_myosotis* | -3.03 | 1.13 | 0.01 | 0.78 |
| *s__Pontibacter_burrus* | -3.00 | 0.86 | 0.00 | 0.18 |
| *s__Mesorhizobium_sp._M00.F.Ca.ET.186.01.1.1* | -2.99 | 0.91 | 0.00 | 0.27 |
| *s__Limosilactobacillus_pulli* | 2.97 | 1.27 | 0.02 | 1.00 |
| *s__uncultured_Desulfatiglans_sp.* | -2.96 | 1.17 | 0.01 | 0.88 |
| *s__Myoviridae_sp._ctHMa1* | 2.96 | 1.30 | 0.02 | 1.00 |
| *s__Paenisporosarcina_quisquiliarum* | 2.95 | 0.97 | 0.00 | 0.46 |
| *s__Rhodoplanes_sp._Z2-YC6860* | 2.93 | 0.84 | 0.00 | 0.17 |
| *s__Streptomyces_sp._SP17BM10* | 2.91 | 0.87 | 0.00 | 0.24 |
| *s__Gracilibacillus_ureilyticus* | 2.90 | 1.03 | 0.00 | 0.61 |
| *s__Flavobacterium_chungangense* | 2.89 | 0.60 | 0.00 | 0.00 |
| *s__Pontibacter_roseus* | -2.89 | 0.94 | 0.00 | 0.46 |
| *s__Viridibacillus_unclassified* | 2.87 | 0.67 | 0.00 | 0.01 |
| *s__Paracoccus_lutimaris* | -2.85 | 0.96 | 0.00 | 0.52 |
| *s__Paraburkholderia_tuberum* | 2.82 | 1.01 | 0.01 | 0.62 |
| *s__Morococcus_cerebrosus* | 2.82 | 0.97 | 0.00 | 0.57 |
| *s__Thermosipho_sp._(in:_thermotogales)* | 2.81 | 1.43 | 0.05 | 1.00 |
